# Comparison of Core Muscle Asymmetry Using Spine Balance 3D in Patients with Arthroscopic Shoulder Surgery: A STROBE-Compliant Cross-Sectional Study

**DOI:** 10.3390/medicina58020302

**Published:** 2022-02-16

**Authors:** Hyunjoong Kim, Seungwon Lee

**Affiliations:** Department of Physical Therapy, Sahmyook University, 815, Hwarang-ro, Seoul 01795, Korea; doong18324@gmail.com

**Keywords:** muscle disorder, postoperative pain, shoulder injuries, immobilization, asymmetric limb muscle stiffness

## Abstract

*Background and Objectives*: Joint immobilization after shoulder surgery can cause an imbalance in the periscapular muscles and affect the kinetic chain throughout the body. There is a difference in core muscle stability because of the asymmetry of the lower extremity muscles. However, the difference due to the asymmetry of the upper-extremity muscles has not been studied extensively. The purpose of this study is to investigate the effect of joint immobilization on the symmetry of the core muscles involved in proximal stability for distal mobility. *Materials and Methods*: Fifty-five patients who underwent arthroscopic shoulder surgery participated in this study. Core muscle asymmetry (CMA) was measured using a body tilt device. The evaluation variables were analyzed according to the surgical site based on the direction of the core muscle ratio and core muscle state ratio. *Results*: No differences in CMA were found based on the surgical site (*p* > 0.05). As a result of the additional subanalysis, significant differences in sex and postoperative day were established (*p* < 0.05). CMA was low during the intensive postoperative rehabilitation period. However, sex-related differences were greater in males than in females. *Conclusions*: The clinical results suggest that core muscle training is necessary to reduce CMA during rehabilitation after the immobilization period has elapsed.

## 1. Introduction

Arthroscopic shoulder surgery is a common practice worldwide. The frequency of arthroscopic shoulder surgery increased by 250% from 1996 to 2006 in the United States and 7.5-fold from 2000 to 2010 in the UK [1]. Shoulder joint immobilization is an essential element in the postoperative treatment of shoulder disorders [2]. However, shoulder stiffness is the most common complication, which causes pain and functional limitations [3].

Joint immobilization is a risky process in rehabilitation because the capsule and ligaments are reduced, and the connective tissue adapts to the shortest functional length [4]. Immobilization in postoperative rehabilitation causes weakness, tightness, and imbalance of the shoulder muscles [5]. The core muscles, which are at the center of all kinetic chains, are related to the upper and lower extremity functions [6]. In addition, patients with shoulder pain lack the coordinative ability of the trunk and lower extremities [7]. Thus, if the asymmetrical upper extremity affects the whole body, it is thought to be related to the core muscles.

Studies on the association between upper or lower extremity asymmetry and core muscles are insufficient. Even in previous studies that showed significant improvement in lower extremity asymmetry through core training, core muscle asymmetry (CMA) was not mentioned [8]. In a magnetic resonance imaging study on asymmetrical transverse abdominis and multifidus muscles in runners, the activation of the right-side core muscles was greater in the case of right-handed runners, which decreased as the training age increased [9]. In other words, an activated pattern for the dominant side was observed [9,10].

CMA is related to overuse, and symmetry is related to training age [9,10]. A whole-body tilt device (Spine Balance 3D, Cyber Medic, Iksan, Korea) is able to identify the side that needs training through the evaluation of the CMA, and core training can be personalized [11]. In this study, we hypothesized that functional limitations due to immobilization in rehabilitation after arthroscopic shoulder surgery create asymmetrical movements and that the altered kinetic chains could be related to CMA.

Therefore, the purpose of this study was to investigate the relationship between upper-extremity asymmetry according to the arthroscopic shoulder surgical site and CMA using a whole-body tilt device.

## 2. Materials and Methods

### 2.1. Study Design

This observational cross-sectional study was conducted during the period of November to December 2020, following Strengthening the Reporting of Observational Studies in Epidemiology Statement (STROBE) guidelines [12].

### 2.2. Participants

This study identified 84 potential participants that underwent arthroscopic shoulder surgery at the Better Hospital, Gwangju, South Korea. The participants were further scrutinized based on the inclusion and exclusion criteria.

Patients who had undergone arthroscopic shoulder surgery and had limited motion due to shoulder pain were included. The exclusion criteria were orthopedic surgery or any other medical history pertaining to the lower extremities, neurological history, or intake of related medications, chronic headache or diseases related to the inner ear, cardiovascular diseases that may affect the balance or intake of related medications, acute dizziness and performing other balance training [7].

Participants who fit the previously mentioned criteria were informed about the aim and the details of the study and all of them agreed to participate. The protocol was approved by the Institutional Review Board of the Sahmyook University on 27 November 2020.(2-1040781-A-N-012020117HR). Prior to commencement, this observational cross-sectional study was registered at ClinicalTrial.gov (accessed on 10 January 2022) (NCT04625816).

### 2.3. Sample Size

The reference population size was based on the standard deviation of transversus abdominis thickness in Ulrike et al. [9], and population estimation was performed using a sample size and power calculator (Version 7.12, Institut Municipal d’Investigació Mèdica, Barcelona, Spain). A sample size of 50 randomly selected subjects would be sufficient to estimate with a 95% confidence and a precision of +/− 0.5, a population mean of a value that has been considered present a standard deviation of 1.8 units. A replacement rate of 0% was anticipated. Therefore, we set the number of participants to 55 to increase the accuracy of the results.

### 2.4. Outcomes

The participants’ age, sex, height, and weight were noted. Body mass index (BMI) was calculated using height and weight, and postoperative time was expressed in days. Arthroscopic shoulder surgery was classified according to the surgical site.

**Figure 1:**
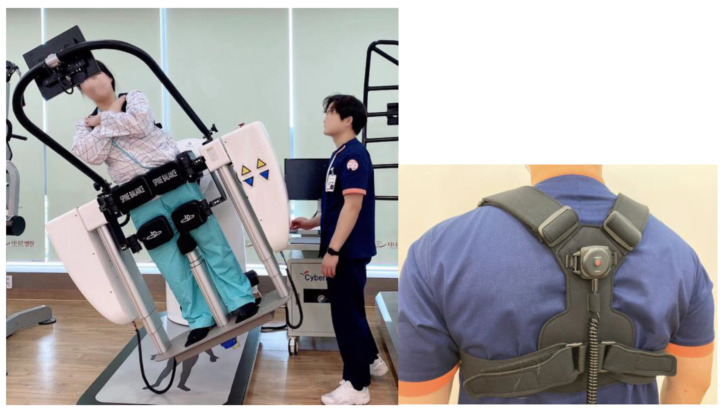
Spine Balance 3D. (**left**) Body tilt device, (**right**) inertia measurement unit (IMU) sensor.

**Figure 2:**
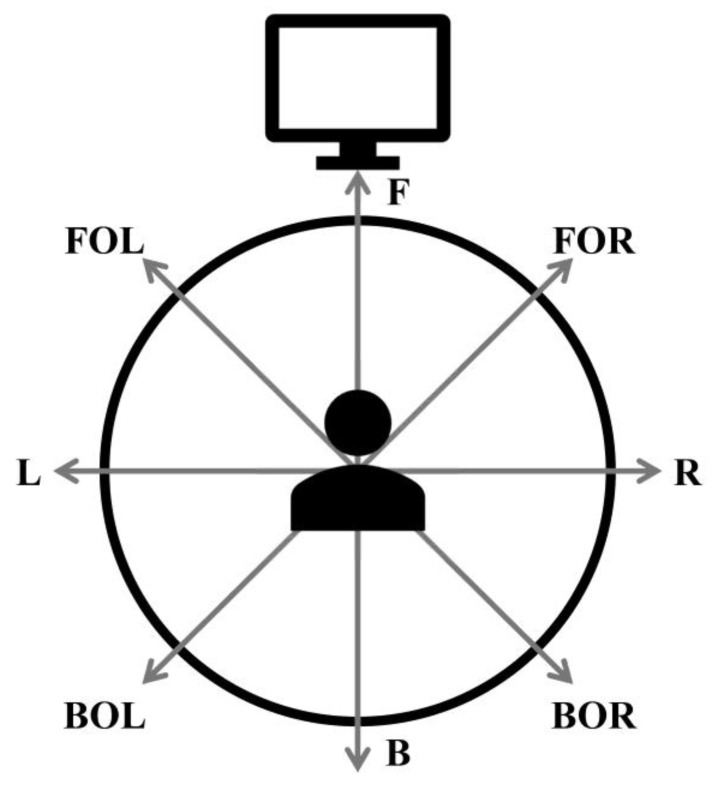
Schematic of the tilting directions of the device. B: back, BOL: back oblique left, BOR: back oblique right, F: front, FOL: front oblique left, FOR: front oblique right, L: left, and R: right.

**Figure 3:**
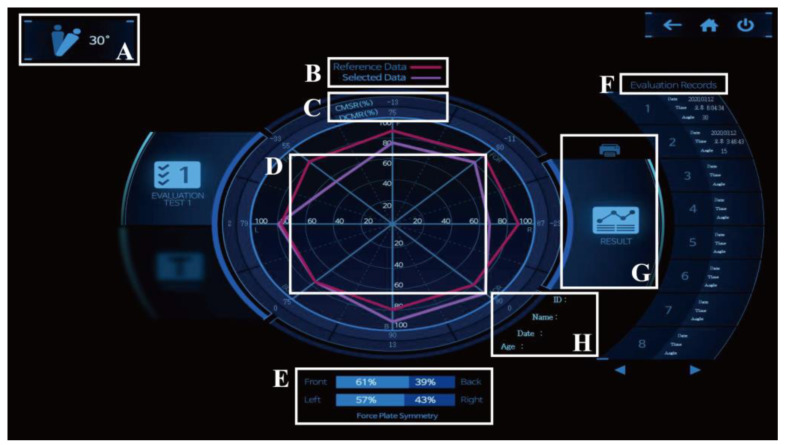
Display interface of a Spine Balance 3D. (**A**) angle setting, (**B**) classification color, (**C**) measured value, (**D**) radial data, (**E**) force plate symmetry, (**F**) evaluation records, (**G**) output result, and (**H**) patient’s information.

In this study, the symmetry of the core muscles was evaluated using a whole-body tilt device (Figure 1). Spine Balance 3D can be used to evaluate the maintenance and maximum strengths of the core muscles and perform a personalized core muscle training program based on the results [13,14,15,16]. In this study, a maintenance strength evaluation of the core muscle was performed. With the device, the core muscles could be tilted up to 60° in eight directions (front (F), front oblique right (FOR), right (R), back oblique right (BOR), back (B), back oblique left (BOL), left (L), and front oblique left (FOL); Figure 2), The angle was set to 15°, which is the setpoint for a patient. After holding the position for 2–5 s in each direction, it returned to the starting position. The results were evaluated by comparing the relationship between the measured data in eight directions and the reference data (Figure 3). In addition, the time and position maintained according to the inclination were measured using an inertial measurement unit (IMU) sensor (Figure 1) with a resolution of 0.1° and a force plate consisting of four vertices under the foot to which the weight was loaded [17].

In this study, the results for the direction core muscle ratio (DCMR), core muscle state ratio (CMSR), and force plate symmetry (FPS) were obtained using a whole-body tilt device. DCMR and CMSR were calculated as a percentage of the time spent in each direction and the position of the IMU sensor with the reference data of healthy adults.

### 2.5. Data Analysis

All statistical analyses were performed using IBM SPSS Statistics for Windows (version 25.0; IBM Corp., New York, NY, USA), and the characteristics of the participants were analyzed using descriptive statistics. Patients were divided into two groups according to the affected side (left or right), and the test of independence was performed using an independent t-test for continuous variables and chi-square test for categorical variables. The normality test was not conducted under the premise that it could be assumed by the central limit theorem. Homogeneity of variance was confirmed using Levene’s test of equal variance. To determine significant differences between the groups, comparisons were performed with independent t-tests.

For further subanalysis, sex, rotator cuff repair, and postoperative day (POD) were compared with the CMA. The differences between the groups of sex and rotator cuff repair were compared using an independent-samples t-test. For CMA analysis according to POD, a one-way analysis of variance test was used for the three groups. The Shapiro–Wilk test was used for normality, and the Tukey HSD test was used for post hoc tests. When the homogeneity of variance could not be assumed, Dunnett’s T3 post hoc test was used. All statistical significance levels (α) were set at *p* < 0.05.

## 3. Results

**Figure 4:**
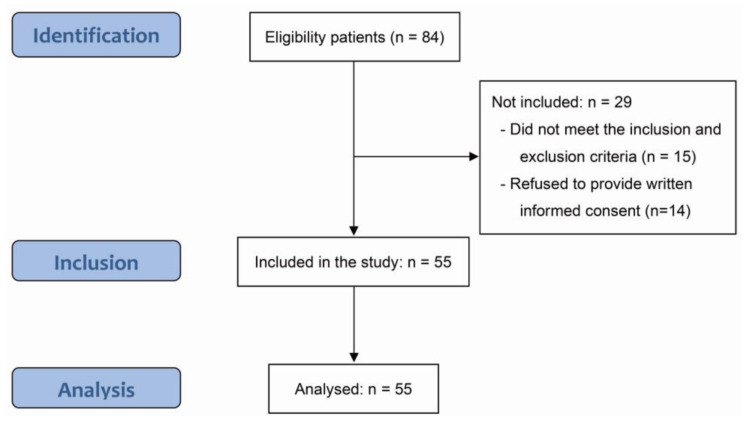
STROBE flow diagram.

In December 2020, among 84 potential participants, as shown in Figure 4, 15 participants who did not satisfy the study inclusion criteria were excluded by the physical therapist. Fourteen participants were excluded because they did not agree to participate in the study. Eventually, 55 patients participated in the study. Table 1 shows the general characteristics of the enrolled participants. Among all the variables, statistically significant differences were found only for height, weight, and capsular release (*p* < 0.05).

### 3.1. Surgical Site

**Figure 5:**
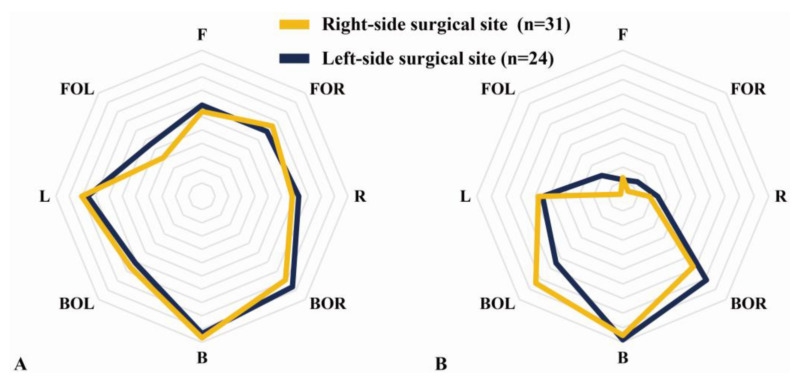
Core muscle asymmetry according to the difference between the right and left sides of the shoulder surgery site. (**A**) Direction core muscle ratio (DCMR). (**B**) Core muscle state ratio (CMSR).

No significant differences were found between the left and right sides in the eight directions (*p* > 0.05). The mean value difference was the largest in the BOL of the CMSR (Figure 5). In addition, no significant differences were found in any of the FPS variables (*p* > 0.05) (Table 2).

### 3.2. Subanalysis of the Differences between Males and Females

**Figure 6:**
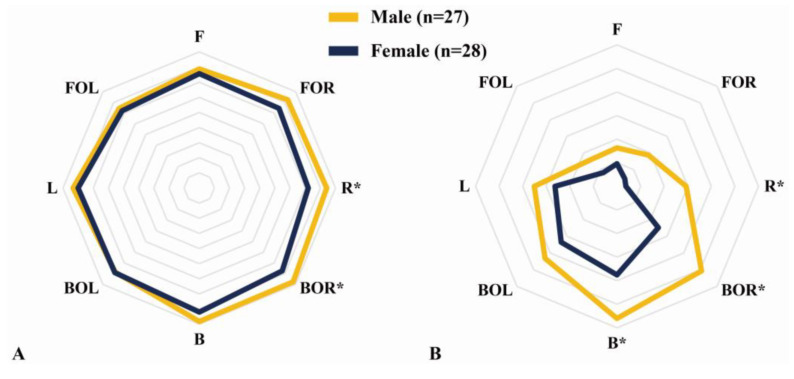
Subanalysis of core muscle asymmetry according to the difference between the males and females. (**A**) Direction core muscle ratio (DCMR). (**B**) Core muscle state ratio (CMSR). Analysis through independent-samples t-tests. * Significant differences between the groups (*p* < 0.05).

The DCMRs in R and BOR were significantly higher in males than in females (*p* < 0.05) (Figure 6A). Similarly, the CMSRs in R, BOR, and B were significantly higher in males than in females (*p* < 0.05) (Figure 6B). However, we found no significant differences in the FPS (*p* > 0.05).

### 3.3. Subanalysis of the Differences between Rotator Cuff and Non-Rotator Cuff Repairs

**Figure 7:**
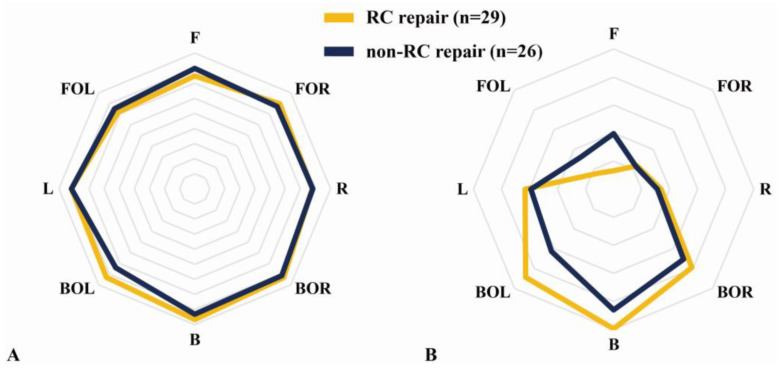
Subanalysis of core muscle asymmetry according to the difference between rotator cuff (RC) repair and non-RC repair. (**A**) Direction core muscle ratio (DCMR). (**B**) Core muscle state ratio (CMSR).

No significant differences in DCMR, CMSR, and FPS were found between the rotator cuff and non-rotator cuff repairs (*p* > 0.05; Figure 7).

### 3.4. Subanalysis according to Postoperative Day

**Figure 8:**
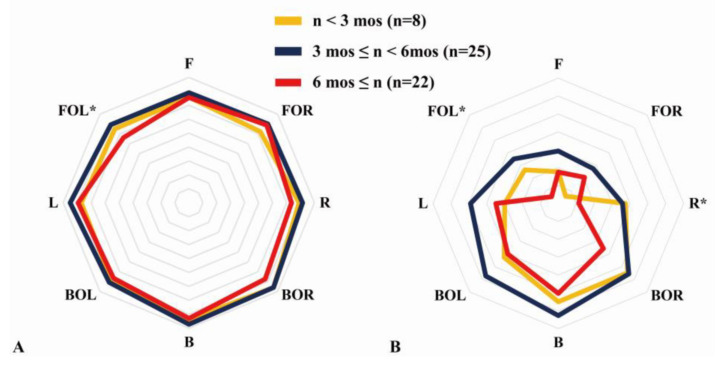
Subanalysis of the core muscle asymmetry according to postoperative day. (**A**) Direction core muscle ratio (DCMR). (**B**) Core muscle state ratio (CMSR). One-way analysis of variance. * Significant difference between the group (*p* < 0.05). mos: months.

The DCMR showed a significant difference in FOL (*p* < 0.05), but no significant difference according to the POD in the post hoc test was observed (*p* > 0.05). CMSR between the R and FOL groups also showed significant differences (*p* < 0.05). As a result of the post hoc test, we found no significant difference in FOL but found a significantly higher CMSR in R at ≥ 6 months than between 3 to 6 months (*p* < 0.05) (Figure 8).

## 4. Discussion

In this study, in DCMR, CMSR, and FPS as variables representing CMA, no significant differences were found according to the shoulder surgical site (*p* > 0.05). Although not through a direct relationship, the relevant literature advocates that control of the spine is achieved through the early activity of the core muscles, such as the control of the trunk during arm movements [18,19,20]. This suggests that it is related to upper-extremity movement and core stability. In addition, it has been reported that the risk of upper-extremity injury increases if the stability of the core is poor [19,21,22]. Therefore, our results may indicate that, in the CMSR compared to healthy adults, negative values of the front, left, and right decreased core muscle endurance, and positive values for the back show that they are dependent on back muscles. However, the absence of a significant difference in a number of results of this study is similar to the findings of previous publications which reported that there was no association between core muscle endurance and shoulder or elbow injuries in middle school baseball players [21,23].

However, significant differences in sex and POD were found in the results of this study (*p* < 0.05). CMA according to sex showed better core muscle symmetry in males than in females. This result is similar to that of a study that analyzed surface electromyography (sEMG) results from the back extensor muscles according to age and sex, and muscle fatigue was observed to be more in females [22]. Core muscle symmetry was best revealed in the period from >3 months to <6 months, which corresponds to the intensive rehabilitation period after arthroscopic shoulder surgery [24,25]. These results can explain the changes in CMA based on training age [9]. In other words, CMA is associated with overuse [11], and it is thought that an intensive rehabilitation period focusing on strength training of the back was effective from 3 months after surgery. In Figure 8, DCMR showed a significant difference in FOL, and, moreover, there was a significant difference seen in FOL and R in the CMSR. These results are consistent with the fact that there was a significant difference overall in the front, based on the fact that the back muscles are activated when tilting to the front [11].

The purpose of this study was to investigate whether the change in the kinetic chain caused by a muscle imbalance in the shoulder joint during immobilization after arthroscopic shoulder surgery is related to core muscle symmetry. By using the evaluation system of a whole-body tilt device, the maintenance strength in the tilting direction, the state of the core muscle as compared with the reference data of healthy adults, and the symmetry of the force plate supporting the weight were evaluated. Thus, the pattern of core muscle asymmetry was examined.

Although there are differences according to the surgical method, after arthroscopic shoulder surgery, patients need joint immobilization for a certain period of time, but this may result in tightness and stiffness in the soft tissues of the shoulder joint [5,26]. This is the cause of the marked increase in compensatory movement rather than the normal movement of the shoulder joint. In patients with rotator cuff tears, the target action shows a higher activation of the large moment arm muscles that play a greater role in motion than the rotator cuff muscles (supraspinatus, infraspinatus, teres minor, and subscapularis), which are known to play an important role in shoulder joint stability. In a related study, the targeting action of the deltoid muscle and adductor (latissimus dorsi) was distinctive [27]. As such, it was predicted that the asymmetric activation of the upper extremity in patients with a rotator cuff injury would affect the asymmetry of the core muscle, but there was no significant association between the symmetry of the core muscle and the affected side.

As in the hypothesis of this study, no specific CMA pattern according to the surgical site appeared, but the accuracy of the evaluation was confirmed through the significant differences seen according to POD and sex. The limitation of this study was that the number of subjects was insufficient to distinguish between the POD of the participants and the period of enrollment in the study. In addition, sEMG was not used for each muscle to demonstrate the characteristics of the CMA.

## 5. Conclusions

The results of this study show that CMA in patients with arthroscopic shoulder surgery is not related to the surgical site but to sex and POD. Our findings suggest that core muscle endurance differed according to sex and was related to training age when the intensive rehabilitation period of patients who underwent arthroscopic shoulder surgery was not considered. However, further studies are needed before conclusions can be drawn on the relationship between the upper extremity and CMA due to joint immobilization.

## Figures and Tables

**Table 1:** The characteristics of the enrolled participants.

Variables	Right-Side Surgical Site	Left-Side Surgical Site	Χ^2^/t	*p*
*N* (%)	31 (56.36)	24 (43.64)		
Age (years)	51.13 (8.78)	50.54 (8.31)	−0.252	0.802
Sex (male/female)	17/14	10/14	1.455	0.228
Height (cm)	167.45 (6.99)	162.63 (8.55)	−2.350	0.023
Weight (kg)	68.74 (13.99)	61.79 (10.50)	−2.029	0.047
BMI (kg/m^2^)	24.32 (3.45)	23.20 (2.07)	−1.406	0.166
Operation (day)	177.10 (69.85)	180.08 (68.59)	0.158	0.875
*Rotator cuff repair*				
Supraspinatus	12	4	9.424	0.093
Infraspinatus	13	1		
Subscapularis	1	5		
*Biceps tenodesis*	24	19	0.024	0.876
*Capsular release*	4	12	9.025	0.003
*ASD*	26	21	0.143	0.705

Values are presented as mean (standard deviation). ASD: arthroscopic subacromial decompression, BMI: body mass index.

**Table 2:** Core muscle asymmetry and force plate symmetry according to the difference between the right- and left-side shoulder surgery sites.

Variable	Right-Side Surgical Site (*n* = 31)	Left-Side Surgical Site (*n* = 24)	t (*p*)	95% CI
*Direction Core Muscle Ratio (%)*				
F	76.77 (2.16)	77.75 (3.49)	0.248 (0.805)	−6.908 to 8.859
FOR	79.00 (2.90)	77.83 (3.00)	−0.276 (0.784)	−9.645 to 7.311
R	77.61 (3.65)	78.58 (2.96)	0.198 (0.844)	−8.860 to 10.801
BOR	81.81 (2.93)	83.29 (2.69)	0.363 (0.718)	−6.722 to 9.692
B	85.35 (2.69)	84.71 (2.67)	−0.167 (0.868)	−8.392 to 7.099
BOL	79.19 (3.49)	78.21 (3.08)	−0.205 (0.838)	−10.616 to 8.645
L	82.13 (2.21)	81.25 (2.69)	−0.255 (0.800)	−7.803 to 6.045
FOL	72.26 (2.87)	75.04 (4.12)	0.571 (0.571)	−6.998 to 12.565
*Core Muscle State Ratio (%)*				
F	−13.45 (2.83)	−13.7 (3.93)	−0.054 (0.957)	−9.722 to 9.208
FOR	−15.00 (3.12)	−13.17 (3.73)	0.379 (0.706)	−7.866 to 11.533
R	−12.32 (3.17)	−11.21 (3.68)	0.230 (0.819)	−8.611 to 10.840
BOR	−2.35 (3.50)	0.21 (3.23)	0.524 (0.602)	−7.244 to 12.371
B	3.06 (3.33)	3.67 (3.21)	0.127 (0.899)	−8.878 to 10.082
BOL	0.84 (2.92)	−3.08 (3.57)	−0.858 (0.395)	−13.090 to 5.246
L	−4.45 (2.53)	−5.00 (2.83)	−0.144 (0.886)	−8.175 to 7.078
FOL	−15.58 (3.35)	−11.96 (4.73)	0.643 (0.523)	−7.675 to 14.920
*Force Plate Symmetry (%)*				
Front	58.26 (1.90)	57.58 (2.07)	−0.239 (0.812)	−6.337 to 4.988
Back	41.74 (1.90)	42.42 (2.07)	0.239 (0.812)	−4.988 to 6.337
Left	48.97 (0.83)	47.83 (1.43)	−0.721 (0.474)	−4.292 to 2.023
Right	51.03 (0.83)	52.17 (1.43)	0.721 (0.474)	−2.023 to 4.292

Values are presented as mean (standard error). B: back, BOL: back oblique left, BOR: back oblique right, CI: confidence interval, F: front, FOL: front oblique left, FOR: front oblique right, L: left, and R: right.

## Data Availability

Not applicable.
